# Chinese expert consensus on echelons treatment of pelvic fractures in modern war

**DOI:** 10.1186/s40779-018-0168-3

**Published:** 2018-06-30

**Authors:** Zhao-wen Zong, Si-xu Chen, Hao Qin, Hua-ping Liang, Lei Yang, Yu-feng Zhao, Representing the Youth Committee on Traumatology branch of the Chinese Medical Association, the PLA Professional Committee and Youth Committee on Disaster Medicine, the Traumatology branch of the China Medical Rescue Association. and the Disaster Medicine branch of the Chongqing Association of Integrative Medicine, Zhao-wen Zong, Si-xu Chen, Hao Qin, Lei Yang, Lin Bai, Jun-qiang Bao, Quan-wei Bao, Jian-mei Chen, Zai-liang Ding, Zhen-qi Ding, Guo-fu Du, De-hao Fu, Shuai Hao, Fei Huang, Jian Huang, Jiang-tao Huo, Wei-dong Jia, Shen Jiang, De-wen Kong, Li-ping Kuai, Nan Li, Wei Li, Xiao-dong Li, Xiao-xue Li, Hua-ping Liang, Guo-dong Li, Peng Liu, Yun-fei Niu, Hao Qin, Ze-wo Qiu, Guo-hui Ren, Yi Shan, Yue Shen, Li-xin Shu, Chen-chao Wang, Zhi-nong Wang, Zhao Xie, Shuo-gui Xu, Xin-zhong Xu, Jia-zhi Yang, Chang-lin Yin, Guan Zhang, Lian-yang Zhang, Lin Zhang, Pei-xun Zhang, Rong Zhang, Guang-yue Zhao, Zhe Zhao, Yu-feng Zhao, Lian-he Zheng, Zhao-wen Zong

**Affiliations:** 1 State Key Laboratory of Trauma, Burn and Combined Injury, Department of War Wound Rescue Skills Training, Base of Army Health Service Training, Army Medical University, ChongQing, 400038 China; 2 First Department, Research Institute of Surgery, Daping Hospital, Army Medical University, Chongqing, 400042 China; 3 Department of Trauma Surgery, Daping Hospital, Army Medical University, ChongQing, 400042 China

**Keywords:** Pelvic fractures, Combat injuries, Classification and treatment, Expert consensus

## Abstract

The characteristics and treatment of pelvic fractures vary between general conditions and modern war. An expert consensus has been reached based on pelvic injury epidemiology and the concepts of battlefield treatment combined with the existing levels of military medical care in modern warfare. According to this consensus, first aid, emergency treatment and early treatment of pelvic fractures are introduced in three separate levels. In Level I facilities, simple triage and rapid treatment following the principles of advanced trauma life support are recommended to evaluate combat casualties during the first-aid stage. Re-evaluation, further immobilization and fixation, and hemostasis are recommended at Level II facilities. At Level III facilities, the main components of damage control surgery are recommended, including comprehensive hemostasis, a proper resuscitation strategy, the treatment of concurrent visceral and blood vessel damage, and battlefield intensive care. The grading standard for evidence evaluation and recommendation was used to reach this expert consensus.

Pelvic fractures account for approximately 3% of all fractures. Those caused by a low-energy impact are mostly stable or mildly unstable fractures without complications of injury to other body parts and can be treated rather easily. In contrast, those caused by a high-energy impact often lead to unstable pelvic fractures, which are prone to complications or comorbidities, such as fatal massive bleeding, organ injuries, and infections, and the mortality rate could be as high as 5 to 20%. In early conflicts, such as the Vietnam War, the incidence of pelvic wounds was relatively low. However, in the wars of Iraq and Afghanistan, an increasing trend in pelvic wounds was observed due to the increased efficiency of fatal weapons and the extensive use of improvised explosive devices, which significantly increase the severity of battlefield injuries. Coupled with the limitations of wartime treatment conditions, it is very challenging to treat combat pelvic wounds. Based on the epidemiology and the latest treatment techniques for pelvic injuries in modern warfare and combined with the current Chinese People’s Liberation Army (PLA)‘s treatment echelon system, we present an expert consensus on the classification and treatment of pelvic fractures in modern war.

The assessment methods that are currently used at specialty hospitals during the war are essentially the same as those employed in time of peace (i.e., non-combat wounds). Therefore, in this consensus, the assessment and treatment methods for combat pelvic injuries at the three treatment echelons prior to early treatment are described. In the “Guidelines for treating war injuries”, which will soon be issued, the existing treatment levels have been adjusted, and emergency treatment is divided into two classes (on-site first aid and early treatment). After these guidelines are issued, this consensus will be adjusted according to the new version of the rules for treating war injuries. It should be noted that war injury treatment is a continuous process; although this expert consensus divides treatments into different levels, the integrity and continuity of patient treatment should be maintained in actual practice.

The evidence and recommendation grades adopted in this expert consensus are mainly based on the standards recommended by the Oxford Evidence-Based Medicine Center and on the criteria commonly used in clinical studies [1–4]. Due to the uniqueness of treating war wounds (e.g., random double-blind experiments are not available), we combined evidence quality grading with the recommendation strength of the “Grading of Recommendations, Assessment, Development and Evaluation” (GRADE) criteria to arrive at a recommendation grade [4]. In this consensus, each recommendation is provided with the evidence and recommendation grades in an “evidence grade/recommendation grade” format.

**Consensus 1**: In modern warfare, a major portion of injuries are from explosive blasts, and the increased severity of the resulting pelvic fractures and the increased proportion of open wounds make these patients prone to fatal massive bleeding, perineal injuries, pelvic organ damage, and traumatic lower limb amputation. Therefore, treatment needs to be converted to a damage control strategy (Grade B/Grade I).

## Overview

Unlike peacetime or previous wars, such as the Vietnam War, the impact from explosive blasts in modern warfare has become a major source of pelvic injuries, imposing a significantly higher fatality rate than gunshot wounds and a significantly increased incidence [5–9]. These injuries have different characteristics than those inflicted in peacetime or in past wars, including the following: 1) Injury severity is significantly increased. In “Operation Enduring Freedom/Operation Iraqi Freedom” (OEF/OIF), the average injury severity score of soldiers with pelvic fractures was 41, whereas that of normal patients with pelvic fractures was only 21–32 [5, 10, 11]. 2) The incidence of open pelvic injuries has increased. The wartime proportion of open pelvic fractures reached 66%, with a significantly higher rate of combined injuries. For example, the co-incidence of urogenital tract injuries, abdominal and pelvic vascular injuries, and rectal injuries was 2.8, 6.5 and 8.5%, respectively [11–14]. 3) Combat pelvic injuries are prone to fatal massive bleeding. Morrison et al. [15] found that in OEF/OIF, severe pelvic fractures were a main cause of uncontrolled massive bleeding, leading to a mortality rate of 85.5%. 4) The incidence of combined perineal injuries is rather high. The incidence of perineal injuries derived from pelvic fractures is normally approximately 0.05% but rose to 2.8% in the Vietnam War and to 5.4% in OEF/OIF, with the incidence of pelvic fractures complicated by perineal injuries as high as 2.8% [8, 16]. 5) The incidence of traumatic lower limb amputation has increased. Due to the widespread use of improvised explosive devices and landmines, the incidence of pelvic fractures combined with traumatic lower limb amputations has risen dramatically. Thus, traumatic lower limb amputation was a characteristic injury of OEF/OIF and is challenging to treat [17, 18].

The above mentioned changes in the characteristics of pelvic injuries have mandated the development of new, distinct requirements, such as the need to focus on treating massive bleeding, co-incident organ damage, and perineal injuries. They have further highlighted the need for the use of more damage control surgery (DCS) concepts for recovery and surgical treatment in combat zones [14, 19].

**Consensus 2**: For battlefield treatment, the Massive hemorrhage, Airway, Respiration, Circulation and Hypothermia (MARCH) method is recommended to rapidly evaluate the injury and determine and treat life-threatening conditions, such as massive bleeding, hemorrhagic shock, airway obstruction, tension pneumothorax, and unstable pelvic fracture, after which the wounded patient need to be quickly evacuated for emergency care (**Grade B/Grade I**).

**Consensus 3**: The presence or absence of pelvic fractures in the wounded need to be comprehensively diagnosed based on the injury mechanism, the presence of lower limb rotation, and localized pain (**Grade B/Grade IIa**).

**Consensus 4**: It is not recommended to apply the pelvic compression-separation test to determine the presence or absence of a pelvic fracture in the wounded (**Grade B/Grade III**).

**Consensus 5**: In the case of pelvic fracture combined with traumatic lower limb amputation, a tourniquet should be promptly applied to control bleeding. For perineal soft tissue bleeding, hemostatic dressings and pressure bandages should be applied to the wound (**Grade B/Grade I**).

**Consensus 6**: For patients suspected of having pelvic fractures, a triangular scarf should be used to bind the pelvis for temporary stabilization. When conditions permit, the use of a pelvic bandage can be more effective. If neither a triangular scarf nor a pelvic bandage is available, other on-hand materials, such as a bed-sheet, bean bag, or many-tailed bandage, can be used to circularly dress and temporarily repair the pelvis (**Grade B/Grade IIa**).

**Consensus 7**: For pelvic fracture patients in hemorrhagic shock, it is recommended to initiate battlefield fluid resuscitation when conditions permit. For resuscitation, blood products such as concentrated red blood cells, hypertonic saline and hydroxyethyl starch are recommended (**Grade B/Grade IIa**).

**Consensus 8**: For open pelvic fracture injuries, oral antibiotics should be administered during the battlefield first aid phase to reduce the risk of infection. Moxifloxacin is generally recommended at a dose of 400 mg (**Grade B/Grade IIa**).

**Consensus 9**: Oral painkillers or intramuscular morphine injections may be given to patients in significant pain (**Grade B/Grade IIa**).

## Battlefield first aid for combat pelvic injuries

First aid is usually performed by the medical unit at or below the battalion level and is generally implemented within 10 min of injury. The focus of battlefield on-site treatment is to follow the principles of advanced trauma life support (ATLS) to quickly assess the condition of the wounded and to diagnose life-threatening conditions such as massive bleeding, hemorrhagic shock, airway obstruction, tension pneumothorax, and unstable fractures. The medical team will then rapidly treat life-threatening conditions such as massive bleeding and airway obstructions, and stabilize pelvis quickly and efficiently, then evacuate the wounded as soon as possible.

### Battlefield injury evaluation

In the battlefield first aid stage, the wounded should be evaluated with priority given in the order of “Airway, Breathing, Circulation, Disability, Exposure and Environment” (“ABCDE”) based on the principle of ATLS. Life-threatening conditions, including the presence or absence of airway obstruction, tension pneumothorax, massive bleeding and hemorrhagic shock, and nerve injury should be diagnosed rapidly. However, massive hemorrhage on the battlefield is the leading cause of preventable war casualties and is much more common than other causes of death, such as airway obstruction. The US army recommends prioritizing the evaluation of battlefield injuries according to “MARCH”: “M” refers to massive hemorrhage, “A” is equivalent to the “A” (Airway) in “ABCDE”; “R” (Respiration) is equivalent to the “B” (Breathing) in “ABCDE”, “C” is equivalent to the “C” (Circulation) in “ABCDE”, and “H” refers to hypothermia [20].

During the on-site first aid stage, the presence or absence of hemorrhagic shock must be determined so that fluid resuscitation can be initiated as early as possible, if necessary, thereby improving the treatment rate of the wounded. By analyzing the Joint Theater Trauma Registry (JTTR) of OIF/OEF, the US army recommended the following criteria for war injury shock [21]: for case without head trauma, if the wounded presents abnormal consciousness and cognition and/or a significantly increased radial pulse frequency of 120 times/min, above, or weakened, even without radial pulse, it should be diagnosed as a shock.

The pelvic compression-separate test is not recommended for the field detection of pelvic fractures, as it can lead to the displacement of unstable pelvic fractures and massive bleeding. The presence or absence of a pelvic fracture in the wounded should be quickly determined via a comprehensive method based on the injury mechanism, the presence or absence of lower extremity rotation and the presence of localized pain. It is not mandatory to evaluate the stability of pelvic fractures on the battlefield [22]. In the case of a suspected pelvic fracture, temporarily fixing and stabilizing the pelvis before rapid evacuation should be performed in accordance with the following methods.

### Hemostasis and bandaging of massive bleeding

Modern warfare, especially with the use of improvised explosive devices, leads to a very high incidence of pelvic fractures co-incident with perineal soft tissue injuries and/or traumatic lower extremity amputations [8, 17, 18], which are all prone to be fatal and bring massive bleeding. In the case of a traumatic amputation of the lower extremity, hemostasis should be used promptly to stop the bleeding; in the case of perineal soft tissue hemorrhage, a hemostatic dressing can be used to pack the wound to stop the bleeding and can serve as a pressure bandage [23–26].

### Temporary stabilization of pelvic fractures

If the wounded is suspected of having a pelvic fracture, temporary stabilization measures should be immediately taken to stabilize the pelvis and reduce bleeding. Triangle scarves are included in the current military first-aid kit, several of which can be connected to each other to form a circular ring and bind the pelvis for temporary stabilization. Pelvic fixation is not required for patients with no possibility of a pelvic fracture based on the injury mechanism, stable hemodynamics, and a normal Glasgow Coma Scale (GCS) score.

A large amount of clinical and war-injury treatment data shows that a variety of commercially available pelvic binders, such as the trauma pelvic orthotic device (T-POD) and the combat trouser binder (CTB), can control pre-hospital severe pelvic bleeding and should be used as soon as possible. If conditions permit, the pelvis of the wounded should be fixed by using a pelvis binding belt prior to evacuation [27–30]. In general, the pelvic banding belt should be easy to use and maneuver without causing an additional injury or affecting subsequent imaging and surgical procedures. The pelvic binding belts that are currently available on the market do not significantly differ. It should be noted that the use of a pelvis binding band in an emergency setting may compress the greater trochanter and the sacrum, thus increasing the risk of a local decubitus ulcer, and should be replaced with external fixators to reduce complications [31].

When triangle scarves and pelvic binding belts are unavailable, on-hand materials such as bedsheets, bean bags, and many-tailed bandages can be used to apply an annular dressing and temporarily fix the pelvis.

### Battlefield fluid resuscitation

The experiences of the North Atlantic Treaty Organization forces, including the U.S. military, have shown that battlefield initiation of fluid resuscitation reduces the incidence of and mortality from multiple organ dysfunction [32]. After controlling enemy fire and evacuating the wounded to a shelter, a venous or intraosseous infusion channel can be established to begin fluid resuscitation. The most commonly used resuscitation fluids are hypertonic saline and plasma substitutes; where possible, blood products such as concentrated red blood cells and fresh frozen plasma may be used [33, 34]. O’Reilly et al. [34] retrospectively evaluated the effectiveness of transfusions during patient transfer to a field hospital among 1592 wounded soldiers following severe trauma who were admitted to a field hospital in Afghanistan from 2006 to 2011. They found that the pre-hospital infusion of blood products reduced the incidence of coagulopathies and the mortality of patients following severe trauma.

### Oral antibiotics and analgesics

For patients with open pelvic fractures, oral antibiotics should be administered on site to reduce the incidence of infections. Moxifloxacin is generally recommended at a dose of 400 mg [35–39].

When the wounded is in significant pain, painkillers can be given orally, or morphine can be injected intramuscularly. Oral painkillers generally include cyclooxygenase-2-specific inhibitors, such as celecoxib and etanercept, which have few side effects on the central nervous system. Morphine is the most commonly used pre-hospital analgesic, and many international emergency medical organizations consider it to be safe and effective for treating pain. US pediatric emergency medical organizations recommend the use of morphine sulfate as an analgesic for treating children who have pain from trauma and the use of naloxone to antagonize its various side effects. The use of morphine sulfate to treat severe pain caused by conditions such as combat fractures and burns is still a gold standard. Intravenous injection is generally recommended because it takes effect very rapidly (in only a few minutes) and because the dose is easily controlled. However, it is often difficult to establish venous access under combat conditions, and therefore, an intramuscular injection may be used, although intramuscularly injected morphine takes effect rather slowly (in 30–60 min) [33, 40].

### Fast evacuation

Frequent moves should be avoided for a wounded patient with a pelvic fracture. After an appropriate halt and stabilization, these patients should be prioritized for evacuation for further treatment.

**Consensus 10**: In the emergency treatment unit, patients with emergency injuries such as massive bleeding, airway obstruction, and hemorrhagic shock may be evaluated sequentially according to the MARCH method (**Grade B/Grade IIa**).

**Consensus 11**: In the emergency treatment unit, in cases of imperfect hemostasis and fixation, additional dressings, fixation and anti-shock therapies are needed (**Grade B/Grade I**).

**Consensus 12**: In the case of a severe pelvic fracture with massive bleeding, the first dose of 1 g tranexamic acid should be administered within 1 h of the injury, and it should be followed by 8 h of a continuous infusion of 1 g tranexamic acid (**Grade A/Grade IIa**).

## Emergency treatment of combat pelvic perineal wounds

Emergency treatment of combat pelvic perineal wounds is usually performed by the medical unit at the regiment (brigade) or equivalent level within 3 h of injury. Emergency treatment is a continuation of battlefield treatment, the main procedures of which include further examination and evaluation of the wounded, additional dressing and fixation methods, and further anti-shock treatment.

### Secondary evaluation

At this treatment level, the main goals of evaluation are to identify injuries in need of emergency treatment, such as massive bleeding, airway obstructions, hemorrhagic shock, and damaged major blood vessels that require a temporary shunt. The MARCH method may still be used to evaluate the wounded.

### Further stabilization of the pelvis

The reliability of the clinical stabilization of the pelvis performed on the battlefield during the first-aid stage in the field should be examined. If it is unreliable, additional triangle scarves and straps should be used to further stabilize the pelvis without removing the original fixators.

### Further improvement in hemostasis

In cases of uncontrolled bleeding, continued strategies to improve hemostasis, e.g., using additional tourniquets and hemostatic dressings, should be employed. In the meantime, in the case of a severe pelvic fracture, especially in patients with multiple injuries and massive bleeding, tranexamic acid should be used as early as possible. It is recommended that the first dose of 1 g tranexamic acid be given within 1 h of the injury, followed by a continuous intravenous infusion of 1 g for 8 h [41, 42]. After analyzing the JTTR database of OIF/OEF, Howard et al. [43] found that tranexamic acid increases the risk of pulmonary embolism and deep vein thrombosis, suggesting that its safety requires further evaluation. In a study organized by the World Health Organization (WHO), 40 WHO members participated in a multicenter randomized double-blind controlled experiment on the effects of tranexamic acid in patients with severe trauma. The study included 20,211 patients with a severe traumatic hemorrhage; 10,096 received tranexamic acid, and 10,115 patients served as controls. The amount of bleeding and the mortality rate of the tranexamic acid group were significantly lower than those in the control group; with respect to embolic events, blood transfusions, and the need for additional surgical treatment, the two groups had no significant differences [44]. Therefore, in general, tranexamic acid is safe and effective for patients with severe pelvic fractures.

### Continued fluid resuscitation

The emergency treatment units of the PLA have been supplied with blood products. In hemorrhagic shock, resuscitation should consist of combining blood products with crystalloids or colloids. For a detailed resuscitation strategy, please refer to Consensus 17.

**Consensus 13**: For patients with combat pelvic injuries, indications for the implementation of a damage control strategy include 1) severe organ injuries with a macrovascular injury, 2) multiple severe injuries, 3) massive blood loss, 4) hypothermia, acidosis, and coagulopathy, and 5) not meeting the threshold values for the above indicators but having an estimated wait time for surgery > 90 min (**Grade B/Grade IIa**).

**Consensus 14**: The main contents of DCS of severe combat pelvic injuries include comprehensive hemostasis measures, an appropriate resuscitation strategy, treatment of concurrent organ and vascular injuries, and combat zone intensive care (**Grade B/Grade I**).

**Consensus 15**: Depending on the specific circumstances of the injury, various measures, such as external pelvic fixators for pelvic stabilization, retroperitoneal packing, bilateral hypogastric artery ligation, and surgical treatment of the damaged organs, can be used to control massive pelvic hemorrhage (**Grade B/Grade IIa**).

**Consensus 16**: Prior to controlling bleeding, it is recommended that a “restrictive hypotensive fluid resuscitation” strategy be implemented, in which fluids are used to resuscitate to a mean arterial pressure of approximately 70 mmHg (**Grade B/Grade IIa**).

**Consensus 17**: In the early treatment unit, it is recommended that those with severe pelvic fractures and massive blood loss be prioritized for transfusion with red blood cells: fresh frozen plasma: platelets at a 1:1:1 ratio. In the case of insufficient blood products, whole blood collection should be organized, and whole blood transfusion should be performed (**Grade A/Grade I**).

**Consensus 18**: If red blood cells, fresh frozen plasma and other blood products or whole blood are unavailable, DP may be an alternative resuscitation material (**Grade B/Grade IIa**).

**Consensus 19**: If red blood cells, fresh frozen plasma and other blood products, and whole blood or DP are unavailable, hydroxyethyl starch may be used as a resuscitation fluid (**Grade B/Grade IIa**).

**Consensus 20**: In the case of combat pelvic injuries with a rectal injury, a colostomy should be performed, and the peritoneal cavity should be thoroughly cleaned to prevent an infection (**Grade B/Grade IIa**).

**Consensus 21**: In the case of combat pelvic injuries with a urethral injury, a bladder ostomy should be performed, followed by repair of the damaged urethra in Stage 2. If a bladder injury is suspected or diagnosed, emergency surgery should be performed to examine and repair the bladder (**Grade B/Grade IIa**).

**Consensus 22**: In the case of combat pelvic injuries with testicle and/or epididymis injuries that may affect reproduction, it is recommended that sperm be retrieved and preserved before debridement (**Grade B/Grade IIa**).

**Consensus 23**: In the case of combat pelvic injuries with a perineal and/or buttock soft tissue injury, a colostomy is recommended only when the external anal sphincter is damaged or if the small intestine is injured. If external anal sphincter function is intact, a colostomy can be omitted, although multiple debridements and vacuum-sealing drainage coupled with an intrarectal catheter are recommended to effectively prevent an infection (**Grade B/Grade IIa**).

**Consensus 24**: As part of the battlefield DCS strategy, in the case of combat pelvic injuries with a lower limb traumatic amputation, traumatic amputees with serious injuries should receive an early amputation instead of attempting limb salvage (**Grade C/Grade IIa**).

**Consensus 25**: Battlefield intensive care of a patient with pelvic injuries should be emphasized. After the vital signs of the wounded stabilize, the patient should be promptly delivered to the nearest treatment center for further management (**Grade B/Grade I**).

## Early treatment of combat pelvic injury

Early treatment of pelvic fractures is generally performed by the medical unit at the division level or its equivalent, usually within 6 h of injury. As mentioned above, pelvic fractures on the battlefield have a rather high incidence and are prone to be co-incident with other, often very severe injuries in various parts of the body, such as the genitourinary tract, pelvic vessels, and rectum, and they can lead to hemorrhagic shock that would require a DCS strategy [11–14]. The peacetime DCS strategy is as follows: patients with severe trauma under the physiological limit are first treated with early-stage simplified surgery and are definitively treated after the patient’s physiologic disorders are properly corrected, after which the patient’s general condition improves. However, the wartime DCS strategy differs in many aspects. For example, it often involves multiple independent treatment units, multiple physicians, multiple resuscitation and stabilization processes, and helicopter and fixed-wing aircraft transport, and therefore, it is essential to ensure the smooth implementation of DCS in wartime [45, 46]. At the same time, due to the wartime conditions and the limited available treatment measures, the main components of DCS for severe combat pelvic injuries include comprehensive hemostasis measures, appropriate resuscitation strategies, the treatment of concurrent organ and vascular injuries, and battlefield intensive care.

### Evaluation and initial diagnosis

In wartime early treatment units, the condition of the wounded can be diagnosed rather accurately by considering the injury mechanism, medical history, physical examinations, and laboratory and imaging analyses. Among them, the early treatment units of the PLA are equipped with ultrasound and X-ray. Ultrasound has several advantages, such as being fast, convenient, noninvasive, and portable, all of which make it feasible for a bedside check. Ultrasound may therefore avoid additional damage to the wounded due to movement and may be very helpful for identifying the presence or absence of co-incident pelvic or abdominal organ injuries [47]. Regarding laboratory tests, the early treatment units of the PLA can perform blood tests, clotting phase analyses, and blood gas analyses, to determine whether a patient has a coagulation disorder and/or acidosis [4, 48]. In addition, the coagulation status of the wounded can be monitored using a thromboelastogram, which is more accurate than conventional coagulation tests and is capable of dynamically monitoring the formation of thrombosis, platelet function, and fibrinogen and fibrinolysis abnormalities. Compared with conventional coagulation tests, thromboelastography is faster, can accurately identify which step of the coagulation pathway is causing problems, and provides coagulation and fibrinolysis information in real time [49–51]. Currently, the early treatment units of the PLA are not yet equipped with a thrombelastograph. However, given its importance in evaluating the coagulation function of the wounded, it is expected that it will be supplied to the early treatment units.

For pelvic fractures, early treatment units also need to focus on evaluating those patients who require DCS. The indications for damage control surgery are currently considered to include 1) severe organ damage combined with a vascular injury, 2) multiple severe injuries, 3) massive blood loss, 4) hypothermia, acidosis and coagulopathy, and 5) having values above the threshold indicators but an estimated waiting time for surgery of > 90 min [52–54].

### Choosing the appropriate hemostasis measure according to different injury conditions

Because pelvic fractures are often co-incident with other life-threatening traumas, in cases of hemodynamic instability, it is necessary to evaluate the abdomen, chest and other potentially injured areas and to examine all possible sites for massive bleeding. After excluding the possibility of massive bleeding in the chest and abdomen, the presence or absence of pelvic hemorrhage needs to be focused on and evaluated.

Correct hemostasis measures can only be found after identifying the source of pelvic fracture bleeding. Under general conditions, the sources of pelvic fracture bleeding include the following: 1) The fracture site. Cancellous bone, which constitutes the pelvic ring, has a rich blood supply. Its continuous or repeated bleeding is the main source of pelvic fracture bleeding. 2) Intravenous and venous plexus bleeds. The two cognominal vessels that accompany the intra-pelvic artery and multiple pelvic plexuses have thin and vulnerable vascular walls. Because contraction of the ruptured veins is rather poor and the structure of their surrounding tissues is soft, it is difficult to produce the pressure required to achieve hemostasis, and therefore, the damaged veins are another important source of bleeding. 3) The internal pelvic artery. The arterial wall is thick and elastic, and therefore, the probability that arteries will cause massive bleeding in pelvic fractures is low. Arteriographies or autopsies have confirmed that the arteries only account for 2.4 to 18.0% of bleeds after pelvic fracture. However, when an arterial rupture occurs, the bleeding will be massive and can be life-threatening. 4) The pelvic wall soft tissue and internal pelvic organs. Pelvic fractures combined with a subcutaneous injury, massive fascia stripping or an internal pelvic organ injury often bleed profusely. The commonly used hemostasis methods for these bleeds include anti-shock pants, external pelvic fixators, arteriography and embolization, internal iliac artery ligation, and compression hemostasis by packing the pelvic cavity with gauze pads [55–57].

**Fig. 1:**
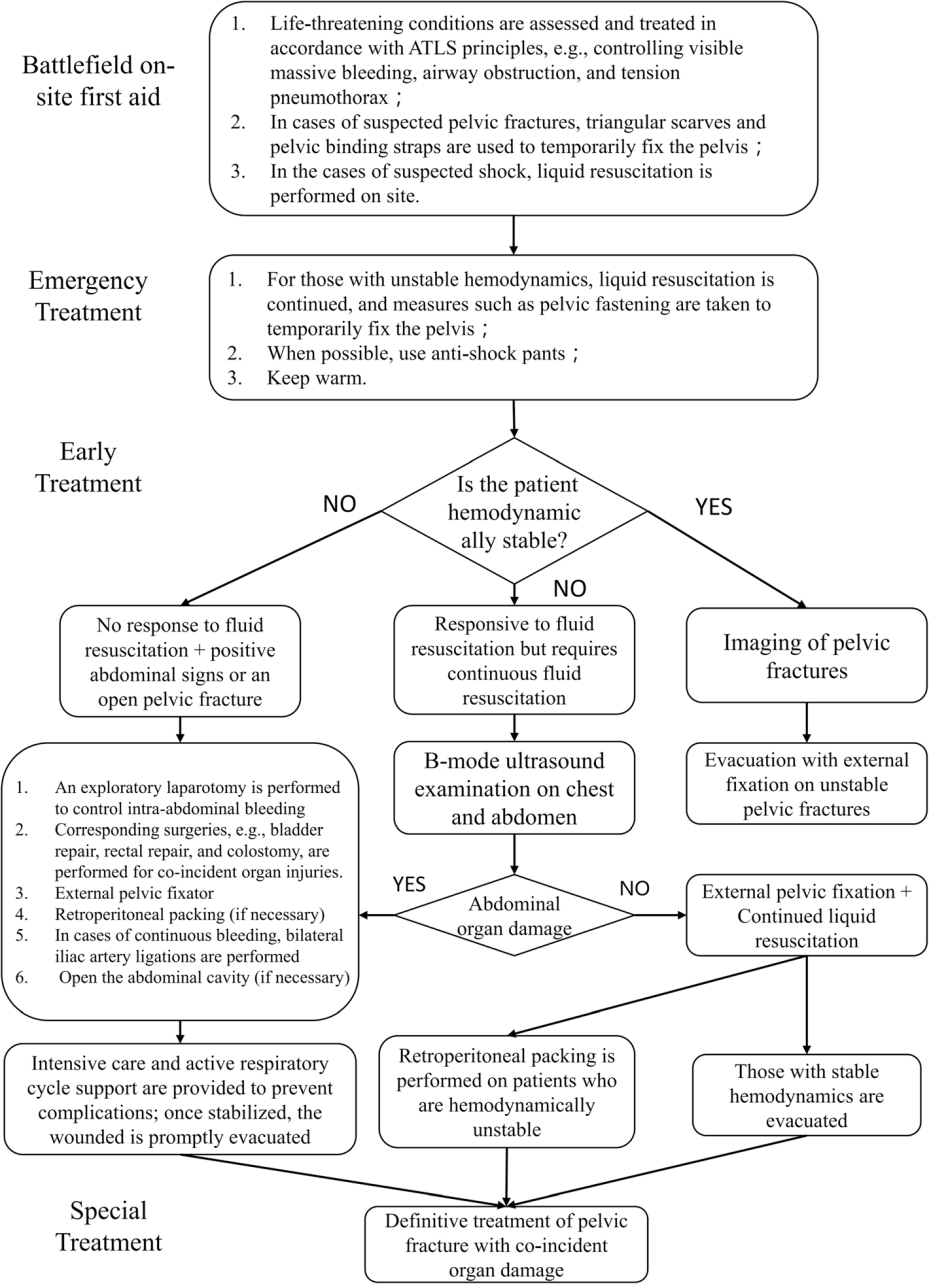
Treatment procedures for pelvic fractures in modern war. The treatment process is designed based on the current treatment level and the equipment in each of the medical units of the PLA. It will be changed accordingly with their development. ATLS. Advanced trauma life support

In a brigade-level medical unit or a field medical clinic, it is necessary to choose an appropriate hemostasis method based on the available instruments, medicine and equipment. Since the brigade-level medical units and field medical clinics of the PLA are not equipped with arteriography-related devices, the potential hemostasis measures include external pelvic fixators, retroperitoneal packing, internal iliac artery ligation, and surgical hemostasis of the damaged organs. We therefore suggest that under the existing conditions, hemostasis of a pelvic hemorrhage should be performed according to the flowchart shown in Fig. 1, with the specific procedures outlined in the following sections.

#### External pelvic fixator

Currently, the commercially available external pelvis fixators can be categorized into two main types: anterior ring fixators and posterior ring fixators. In wartime, the former is more practical to use [58, 59]. Mathieu et al. [59] reported the experience of the French army in using an external pelvic fixator in the 2004–2009 OIF/OEF as a measure for damage control resuscitation (DCR). Eighteen patients required an external pelvic fixator. The external pelvic fixator was kept in some patients until the fracture healed, whereas the external pelvic fixator needed to be replaced with an internal fixator in some patients. None of the patients had an infection.

#### Retroperitoneal packing

For pelvic fracture patients with a retroperitoneal rupture, the stuffing effect after a retroperitoneal loss is prone to fatal massive bleeding that is not controllable with conventional hemostasis methods; in this case, retroperitoneal packing can effectively control the bleeding [55, 60, 61]. Two surgical approaches may be used [62]: in the case of visceral rupture or the need for an examination, a rectus incision can be made and extended downward to the symphysis pubis. In the absence of examination indications, a transverse incision over the symphysis pubis can be made without opening the peritoneum, thus permitting the peritoneal hematoma to be exposed from the front and allowing blood and blood clots to be removed. The bladder is pulled laterally, and the pelvic rim is carefully probed and manually separated, taking care to avoid tearing any blood vessel branches between the iliac and obturator vessels. The posterior is examined to the greatest extent possible along the edge of the pelvis, and wet gauze pads with fluoroscopy markings are sequentially packed into the pelvis by being inserted downward and toward the posterior using a rounded pincer clamp. In general, the first wet gauze pad is placed in the deepest spot, below the sacroiliac joint; the second is placed in the middle of the pelvic fossa, in front of the first pad; and the third is placed in the retropubic fossa that is posterior and lateral to the bladder until it is solidly packed. After completing the packing at one side, the bladder is pulled to the opposite side so the packing can be similarly performed on this side. Generally, 5 or 6 pieces of 25 cm × 25 cm wet gauze pads are needed. After the packing, the wound is washed and continuously sutured layer by layer; the packing is removed 48–72 h after the operation to prevent infection. Arul et al. [63] found that extraperitoneal packing in combination with the use of absorbable hemostatic gauze loaded with chitosan can control bleeding, with neither significant adhesions nor a remarkable residual.

#### Internal iliac artery ligation

When the above method is ineffective, bilateral internal iliac artery ligation can be chosen to help control the bleeding [55, 56]. There are two surgical approaches to internal iliac artery ligation: transabdominal ligation and transperitoneal ligation.

#### Surgical treatment of damaged organs

When the clinical symptoms, signs and B-mode ultrasound examination reveal combined organ damage, an exploratory laparotomy should be rapidly performed to treat the damaged organs and control the bleeding. Details of the treatments for damage to various organs are described later.

### Damage control resuscitation

#### Restrictive (hypotensive) fluid resuscitation

Pelvic fractures are often combined with organ damage, and when the bleeding from the organ damage is not effectively controlled, “delayed fluid resuscitation”, also known as “restrictive (hypotensive) fluid resuscitation” is recommended. In particular, in the case of a thoracotomy for a cardiac vascular injury, too much or too rapid rehydration can be harmful, and in case of a cardiac tamponade, a large amount of fluid supplementation cannot increase the cardiac output but can induce fatal bleeding due to increased intra-cardiac pressure and flushed clots, which can lead to the correct time of surgery being missed. If the radial artery pulse is palpable and the systolic blood pressure is approximately 90 mmHg (1 mmHg = 0.133 kPa), rehydration can be omitted before bleeding is controlled. If the radial artery pulse is weak or non-palpable and the blood pressure is much lower, equilibrium fluid of an appropriate amount may be supplemented. If the radial artery pulse disappears and then resumes, fluid resuscitation may be temporarily postponed or suspended under close monitoring [64–66].

When considering fluid resuscitation of pelvic fracture patients in shock, it is recommended to not use an excessive amount of vasoconstrictor drugs, which are used only if the patient cannot maintain their blood pressure even after sufficient fluid resuscitation. It is appropriate to maintain the patient’s blood pressure at a low normal level to avoid aggravating the massive loss of blood components caused by bleeding, thereby worsening the condition.

#### Choice of resuscitation liquid type and proportion

In early treatment units, a red blood cell:freshly frozen plasma:platelet ratio of 1:1:1 is recommended for severe pelvic fracture patients with massive blood loss [67–70]; if there are insufficient blood products, whole blood collection may be organized and transfused instead [71]. DP can be stored at 2–35 °C for 15–24 months while maintaining a clotting activity of 75–100%. Commercially available products currently include LyoPlas and LyoPhil. When blood products such as red blood cells, fresh frozen plasma or whole blood are unavailable, DP can be an alternative resuscitation fluid. DP has been approved for use by the British, French, German and Israeli armies [72] but has not been approved by the FDA. US special forces are equipped with DP made in France. Only when blood products such as red blood cells, fresh frozen plasma, whole blood or DP are unavailable can hydroxyethyl starch be used as a resuscitation liquid [73–75].

### Treatment of a co-incident rectal injury

In modern warfare, the incidence of pelvic fractures combined with a rectal injury is approximately 8.5%. Lower abdominal pain, tenesmus and anal bleeding are important clinical manifestations of a rectal injury. When performing an anus finger exam, presacral tenderness can be observed. Occasionally, a palpable fracture end penetrating the rectum or rectal wall opening leads to visible blood on the glove. If the rectal rupture is above the peritoneal fold, significant peritoneal irritation may be observed. Since the location of the rectum is rather deep, its symptoms may be masked by the clinical symptoms of a posterior pelvic ring fracture or damage to other pelvic organs. Therefore, a sacral fracture patient with anal bleeding or visible blood on the anus finger exam needs to be evaluated for the possibility of rectal damage [39, 76].

When rectal damage is identified, emergency surgery must be performed [76]. A medial abdominal or left medial abdominal approach is typically used to enter the abdominal cavity, remove intraperitoneal contaminations and locate the rupture site on the rectal wall. After trimming, a transverse double-layer suture is applied, followed by a proximal colostomy to divert stool and facilitate wound healing.

### Treatment of a co-incident urethral injury

In wartime, the incidence of a pelvic fracture combined with a urogenital injury is approximately 2.8% [12, 77, 78]. A posterior urethral injury is a common concurrent injury among male pelvic fracture patients. The female urethra is short and thick, and it may be affected by pubic fracture injuries. However, this is rare and is often accompanied by a vaginal injury, which may mask the urethral injury and lead to a missed diagnosis. Urethral bleeding or urethral blood is an important manifestation of a urethral injury, in which the wounded often presents with a distended abdomen and perineal pain, a desire to urinate but an inability to do so, and a B-mode ultrasound revealing a filling level of the bladder. If the urethral catheter cannot reach the bladder, the diagnosis of a broken urethra can be made. In urethral injuries that allow the urethral catheter to enter the bladder, a urethral catheter can be used as a stent for 3 weeks and serve as a non-surgical treatment. For pelvic fracture patients with a completely broken urethra, the following two different approaches have generally been used: urethral realignment and an early cystostomy followed by a urethral prosthesis at an appropriate time [79]. A cystostomy is simple and easy to perform and is thus suitable for wartime damage control [16]. Abdin et al. [80] described a less invasive transdermal ureterostomy that is even simpler than a conventional colostomy. It is therefore suitable for controlling the severe damage caused by a pelvic fracture.

In patients with abdominal pain, the urge but inability to urinate, or a small amount of bloody urine or blood at the urethral opening after the injury, examinations should be performed for peritoneal irritation signs, such as the presence or absence of abdominal tenderness, muscle tension, rebound tenderness, and the weakening or disappearance of bowel sounds. Those with a positive exam require further examination so a clear diagnosis can be made. In the case of a bladder rupture, emergency surgery should be performed to repair the bladder [16]^.^

### Treatment of combat testicular and epididymal injuries

The basic principles of the treatment of the testis and epididymis are the same as those upheld during peacetime, i.e., multiple debridements are applied. However, it should be noted that in patients who have severe testicular damage that is likely to affect their reproductive capacity, it is recommended that sperm be retrieved and preserved prior to the debridement [16].

### Treatment of combat perineal and buttock soft tissue injuries

In the past, it was believed that injuries in the perineal area and buttocks generally required a colostomy to reduce the incidence of infections [8]. However, recent treatment experiences during wartime and peacetime have demonstrated different outcomes. Ramasamy et al. [13] revealed that in a set of combat perineal trauma cases, 82.8% of the wounded suffered from a deep infection during hospitalization. Twenty-five cases were located in the Faringer I region: 9 cases with an ostomic shunt developed a deep infection, and 12 of the 16 cases without an ostomic shunt developed a deep infection. These results show that ostomy fails to reduce the incidence of infections but can lead to many complications, such as intestinal adhesions. It is now believed that a colostomy should be recommended only in those patients with external anal sphincter or small intestine damage. Further, as long as the external anal sphincter function is intact and a complete perianal skin patch is present, an ostomy is unnecessary, and repeated debridement and vacuum-sealed drainage coupled with an internal rectal catheter can effectively prevent an infection [81–87].

### Treatment of co-incident traumatic lower limb amputations

In modern warfare, the incidence of a pelvic fracture combined with a traumatic amputation of the lower limb is rather high. According to Penn-Barwell et al. [17], among the 77 evaluated patients with traumatic lower limb amputations, 17 (22%) had a pelvic fracture. The concurrent rates of a unilateral traumatic lower limb amputation, bilateral traumatic lower limb amputation, or transfemoral traumatic lower limb amputation with a pelvic fracture were 10, 30 and 39%, respectively. There are no absolute criteria for limb amputation or limb salvage. Under normal circumstances, amputation should be considered in cases of destroyed large blood vessels, widespread muscle damage and soft tissue injury, destroyed or damaged major nerves, high lactic acid concentration, or prolonged warm ischemia time. At the same time, the physical damage severity score can help determine whether an amputation is necessary. The US army’s experiences in Afghanistan and Iraq indicate that the integrated use of clinical symptoms, the physical damage severity score, Doppler ultrasound and CT angiography can improve the accuracy of this evaluation [87–90]. In the case of a severe pelvic injury, early amputation should be performed on patients with severe traumatic injury instead of attempting limb salvage as a measure of DCS [17, 18, 91].

### The use of early antibiotics

Staphylococcus aureus and Pseudomonas aeruginosa remain the main causative pathogens of soft tissue wound infections in China. Before obtaining a confirmative drug sensitivity test result, empiric antibiotic therapy should be commenced against these pathogens. After obtaining a confirmative drug sensitivity test result, antibiotics should be selected according to the results. In using antibiotics, the following should be considered: 1) Antibiotics are only auxiliary to surgery as a means of treating wound surface soft tissue infections and should not be abused. Such abuse may induce the emergence of drug-resistant pathogens, leading to greater difficulties for subsequent treatments. 2) The spectra of the pathogenic strains in different regions and environments will change; for example, in field conditions, the possibility of a bacillus infection (gas gangrene) or anaerobic clostridium infection (tetanus) increases, making a tetanus antitoxin injection required for open wounds in addition to the use of an appropriate antibiotic (e.g., penicillin) [39, 92].

### Battlefield intensive care

Intensive care is an important part of the DCS strategy for combat pelvic injuries. As late as the 1990s, an intensive care unit had not been established in field hospitals of the US army, whose approach was to evacuate wounded patients in critical condition as soon as possible [93]. However, in the early stage of OIF, the US army set up an intensive care unit in their field hospitals and adopted a battlefield intensive care model that is centered around the intensive care physician, thereby effectively reducing mortality without increasing the logistical burden and hospital stay of the wounded [93, 94].

## Prospect

In summary, combat pelvic injuries have different characteristics than peacetime injuries and thus require different treatment processes (Fig. 1). Based on the existing treatment concepts and the PLA’s existing treatment echelons, we have developed an expert consensus on the treatment of combat pelvic injuries in modern warfare. The treatment process should be adjusted and updated based on advances in new treatment techniques and concepts, changes in the fatality effects of the weapons of war, and changes in the PLA’s combat unit system. In addition, strong logistical support is a prerequisite for the implementation of the above treatment measures (e.g., transporting blood products during the battlefield emergency treatment stage [95]). It is expected that with the enhancement of the PLA’s military support capabilities, the existing treatment processes will be optimized accordingly.

## Abbreviations

ATLS: Advanced trauma life support
CTB: Combat trouser binder
DCR: Damage control resuscitation
DCS: Damage control surgery
DP: Dried plasma
GCS: Glasgow coma scale
GRADE: Grading of recommendations, assessment, development and evaluation
JTTR: Joint theater trauma registry
MARCH: Massive hemorrhage, airway, respiration, circulation and hypothermia
OEF: Operation enduring freedom
OIF: Operation Iraqi freedom
PLA: People’s liberation army
T-POD: Trauma pelvic orthotic device
WHO: World Health Organization
